# Factors associated with health risk behavior among school children in urban Vietnam

**DOI:** 10.3402/gha.v6i0.18876

**Published:** 2013-01-18

**Authors:** Tran Bich Phuong, Nguyen Thanh Huong, Truong Quang Tien, Hoang Khanh Chi, Michael P. Dunne

**Affiliations:** 1 Center for Health and Community Development, Vietnam Union of Science and Technology Associations, Hanoi, Vietnam; 2 Faculty of Social Science and Health Behavior, Hanoi School of Public Health, Hanoi, Vietnam; 3 Center for Community Health Research, Hue University of Medicine and Pharmacy, Hue, Vietnam; 4 School of Public Health, Queensland University of Technology, Brisbane, Australia

**Keywords:** risk behavior, risk factor, protective factor, school children, Vietnam

## Abstract

**Background:**

Health risk behavior among young people is a public health problem in Vietnam. In addition, road traffic injuries are the leading cause of death for those aged 15–29 years. The consequences can be devastating for adolescents and their families, and can create a significant economic burden on society.

**Objective:**

The aim of this study was to identify protective and risk factors that may influence three health risk behaviors among school children: suicidal thinking (ST), drinking alcohol (DA), and underage motorbike driving (MD).

**Methods:**

A cross-sectional survey of 972 adolescents (aged 12–15 years) was conducted in two secondary schools in Hanoi, Vietnam. The schools were purposely selected, one each from the inner city and a suburban area, from which classes (grade 6 to 8) were randomly selected. All students attending classes on survey days took part in the survey. The anonymous, self-completed questionnaire included measures of risk behavior, school connectedness, parental bonding, and other factors. Multivariable regression models were used to examine associations between the independent variables and the three health risk behaviors controlling for confounding factors.

**Results:**

Young people in the inner city school reported a higher prevalence of all three risk behaviors than those in the suburban area (ST: 16.1% [95% confidence interval, or CI, 12.9–19.3] versus 4.6% [95% CI 2.7–6.5], *p*<0.001; DA: 20.3% [95% CI 16.8–23.8] versus 8.3% [95% CI 5.8–10.8], *p<*0.001, and MD: 10.1% [95% CI 7.4–12.8] versus 5.7% [95% CI 3.6–7.8], *p*<0.01). School connectedness and mother and father care appeared to be significant protective factors. For males, bullying in school was associated with suicidal thoughts, whereas for both males and females, school connectedness may be protective against suicidal ideation.

**Conclusion:**

This study supports findings from other nations regarding suicidal thoughts and alcohol use, and appears to be one of the first to examine risk and protective factors forMD. Health promotion within schools should be introduced to improve students’ feelings of connectedness in combination with communication and education campaigns focusing on parental care and engaging teachers for the promotion of safer, supportive school environments.

It is clear from research worldwide that many children and adolescents experience behavior disorders. There are differences in prevalence estimates across studies, but overall 10–20% of children and adolescents in developed and developing countries have mental and behavioral disorders (1). Substance use, alcohol consumption, smoking, fighting, and suicide attempt were all found to be prominent risk behaviors among the young (2–4). In Asia, the prevalence of major risk behaviors has been found to be high in Bangkok, Thailand (4). Chinese adolescents appear to be at high risk of suicide ideation and suicide attempt (5, 6). Many factors influence risk behaviors in children and adolescents, especially characteristics of families and schools. Support from family equips young people to deal with stressful situations and communication with parents is key in establishing the family as a protective environment (7). Conversely, having poor relationships with parents or experiencing parental divorce or separation is associated with behavioral problems (4). Findings from the Health Behavior in School Aged Children Study (HBSC) show that experiences in school can be crucial to the development of health behavior; in particular, school connectedness is linked with positive health practices (7).

**Policy recommendations**

- More attention from government and different sectors in society is required to tackle risk behaviors among Vietnamese adolescents. Financial resources are needed to gather evidence on mental health and risk behaviors among Vietnamese adolescents and the potentially modifiable causal factors. This requires public health agencies to mobilize budgets from national and international sources to facilitate the research.
- Capacity improvement for health and counseling services, especially at the school level is needed, to help identify health risk behaviors, including ST and behavior among adolescents.
- Collaboration among education, transportation, police, and local government is needed to prevent underage driving of motorbikes.
- Develop specific programs to assess, manage, and prevent young people engaging in health risk behaviors. These intervention programs need to incorporate more than one strategy, such as school-based health education, human relationships education, and bullying prevention, alongside parental involvement, and with very high risk youth, social welfare support is also needed. .
- Community awareness-raising strategies with mass organizations (such as the youth union) and youth-focused media should be implemented with rigorous evaluation.

In Vietnam, there is increasing awareness of youth risk behaviors in the media and in public debate. Recently, results from limited research have shown that a relatively large proportion of Vietnamese children exhibit behavioral problems (8–11). Of the 2,591 adolescents aged between 12 and 18 years who participated in Huong's survey in 2004–2005, 9.2% had thought seriously about suicide in the previous 12 months and 8.7% had consumed alcohol in the past 30 days (10). Nearly half (48.7%) of 515 attempted-suicide patients admitted to the biggest hospital in Hanoi were aged between 15 and 25 (11). Findings from two national population-based surveys, the Survey Assessment of Vietnamese Youth (SAVY) I (2003–2004) and II (2009–2010), show that there was a significant increase in the prevalence of suicidal behavior among Vietnamese adolescents over this time period (from 5.28 to 12.21%). Another serious problem for Vietnamese youth is traffic-related injury, which is a leading cause of death in children and adolescents (12, 13). Seventy percent of the traffic accidents in Vietnam are related to motorcycle crashes, and 88.14% of motorcycle crash-related deaths are due to head trauma (14).

Behaviors established during adolescence often continue into adulthood, eventually resulting in substantial morbidity and mortality (7). Given that youth under 18 years of age account for 30% of the Vietnamese population (15), the mental health and risk behaviors of school children should receive attention and resources from the government and many sectors in society. In this study, we examined the factors that influence three health risk behaviors of school children in Vietnam: (a) thinking about suicide in the past 12 months, suicidal thinking (ST); (b) drinking alcohol (DA) in the past month; and (c) underage motorbike driving (MD) in the past month. Underage driving is a particularly important health risk among Vietnamese adolescents. This study examined these behaviors in two schools located in inner city and suburban Hanoi. The overall purpose was to produce insights that can be used by government and non-government agencies in the design and delivery of programs to prevent harmful behaviors among children and adolescents.

## Methodology

### Study setting

A cross-sectional survey was conducted in two secondary schools in Hanoi city. One school was in one of the four ancient districts of Hanoi, which is also the national political administration center of Vietnam, and the other was in a suburban district that has an average level of socioeconomic development for Hanoi. The purpose of choosing two schools in different locations was to gain some diversity in school characteristics. The bus is the main means of public transport in Hanoi. It was introduced about a decade ago and has not met the transportation needs of the city. In Hanoi, majority of families have at least one motorbike and use it as their main means of transportation. According to Vietnamese Law, children aged below 18 years are not allowed to drive motorbikes; however, given the availability of motorbikes and the weak enforcement of this law, under-aged driving is common in big cities in Vietnam.

Wine and beer are widely available and often served at family parties or weddings. School children in the study locations could buy wine or beer from shops and small kiosks in the areas very easily and at relatively cheap prices.

### Sampling

The study applied one-stage cluster sampling (school class). A total of 972 children aged 12–15 years from the two schools participated in this survey. The schools were purposely selected from the inner city and a suburban area. In the inner-city school, one class consisted of 45–55 children, whereas in the sub-urban school a class consisted of 30–35 children. Thus, 10 out of 30 classes and 15 out of 28 classes were selected from each school for the study, respectively. The total number of classes selected in each school was equally divided across the three grades. Classes were randomly selected within the grades. All students in the randomly selected classes at school on the survey dates took part in the survey.

This study assessed the association between five main independent variables (bullying, parental bonding, school connectedness, inter-parent and sibling conflicts, and self-reported academic achievement) and three health risk behaviors (dependent variables). The questionnaire consisted of 61 items divided into four domains: demographic characteristics, family environment, school environment, and behavior. The following scales and questions were included.

### Health risk behaviors

Three questions asked whether the youth had experienced suicidal thoughts, alcohol consumption, or MD. The questions included dichotomous responses (Yes, No) and were adapted from the Youth Risk Behavior Survey (YRBS) developed by the Centers for Disease Control and Prevention in the United States. The YRBS has been widely used in research with adolescents in Asian countries, such as China, Thailand, and also in Vietnam, and has demonstrated itself as a suitable tool (10). The questions ‘Did you drink wine/beer/other alcohol in the past 30 days?’, ‘Did you drive a motorbike (by yourself) in the past 30 days?’, and ‘Did you ever think seriously about suicide in the past year?’

### Parental bonding

We used the Parental Bonding Instrument developed by Parker et al. in 1979 (16). This scale has been widely used in many projects and is found to have good reliability and validity (in this study, Cronbach's alpha coefficients for this scale equaled 0.83 and 0.84 for mother and father care, respectively). It consists of 25 items to be completed by the respondent about their relationships with their father and mother separately, assessing two dimensions of perceived parental bonding: ‘care’ and ‘over-protection’.

### School connectedness

This scale consists of seven items adapted from the California Healthy Kids Survey 2004 (17). In this study, the Cronbach's alpha was 0.84.

### Bullying exposure scale

This scale was developed by the research team based on an extensive literature review of bullying scales that have been used in Vietnam and other countries. The scale consisted of five items about past-month experiences of bullying and used a three-point response format that included ‘No’, ‘Sometimes’, or ‘Often’. Cronbach's alpha for this scale was 0.79, suggesting good internal consistency.

### Inter-parent and sibling conflicts

Three questions were asked about parental quarrelling, parental fighting, and sibling conflict using a 4-point scale (*Never; Rarely; Sometimes; Often*) (10). *Academic achievement* included a single question, which asked respondents to rate their academic achievement in the previous semester on a 4-point scale (*Excellent; Good; Fair; Poor*).

### Data collection

Anonymous questionnaires were self-completed in classrooms. Before answering the questionnaires, the researchers explained the purpose of the survey and the procedures, and students were asked to focus on their own response without discussion. To protect confidentiality and to ensure standard administration procedures, questionnaires were distributed by researchers in the absence of class teachers. Study participants put the completed questionnaires into sealed envelopes that had been distributed beforehand together with the questionnaires. The questionnaire took 25–30 min to complete and the in-class survey procedure was completed within one normal school session.

### Data management

To ensure data quality, the research team screened all returned questionnaires before starting data entry. Data were entered by experienced research assistants and 10% of the returned questionnaires were randomly selected to verify the data entry.

### Data analysis

SPSS for Windows version 12 was used to analyze the data. Multivariable logistic regression was conducted to examine factors that may influence the *three* health risk behaviors separately by gender. The hierarchical forward stepwise method was used to fit four blocks of independent variables to each outcome. The four blocks represented: (1) demographic characteristics that include age, religion, grade, school location, and economic status; (2) family characteristics that include parent marital status, mother education, mother occupation, father education, father occupation, parent alcohol problems, parent drug problems, and emotional support; (3) family variables that include mother care, mother overprotection, father care, father over protection, parental quarrelling, parental fighting, and sibling conflict; and (4) school variables that include school connectedness, academic achievement, and bullying. The forward stepwise selection procedure determines if a variable statistic significantly contributes to the odds of taking risky behaviors. At the end of the analysis procedure, only significant variables remained in the models.

### Ethical considerations

This research received approval from the Human Research Ethics Committee of Queensland University of Technology (QUT) in Brisbane, Australia, and the Hanoi School of Public Health, Vietnam. The research was also approved by the Health Departments of the two districts and leaders of the two secondary schools. The study applied a ‘passive parental consent’ method. First, the research team worked with the schools and their parent associations to gain agreement for conducting the survey. With help from the parent associations, study information sheets and consent forms were sent to all parents whose child/children had been selected for the study. Parents who did not respond that they would *not* allow their children to participate in this study were considered to have agreed to the study. In fact, no parents of the sampled children actively refused.

## Results

### Description of study participants

Demographic characteristics of the participants are presented in Table 1. Of the 966 students who rated their academic achievement in the last semester, less than 2% reported unsatisfactory performance. With regard to family economic status, a proxy measure related to ownership of means of transportation showed that 12.3% had ‘high economic status’ (family owns a car), 74.1% were at medium level (owned one or more motorbikes), and 13.6% were rated as poor (the family only owned a bicycle). With regard to the family environment, more than 95% of participants reported living with both natural parents. Almost all participants reported that they were Kinh, the most prominent ethnicity in Vietnam. When participants were asked about who they would seek out when they need emotional support, more than a third (36.6%) said they would seek it from friends, 20% from their mother, 14.6% from siblings, and only 4.9% from their father. More than 15% of the participants reported that they would not seek emotional support from anyone (Table 2).

**Table 1:** Demographic characteristics of the participants

Characteristics	%
Region (*n*=972)	
Urban	51.7
Rural	48.3
Sex (*n*=952)	
Male	51.3
Female	48.7
Grades (*n*=972)	
Grade 6	33.3
Grade 7	36.0
Grade 8	30.7
Self-reported academic achievement in the school (*n*=966)	
Distinction	32.8
Credit	44.9
Satisfactory	20.5
Unsatisfactory	1.8
Ethnic group (*n*=960)	
Kinh	99.5
Others	0.5
Religion (*n*=932)	
No	73.9
Yes	26.1
Family economic status (*n*=961)	
High	12.3
Medium	74.1
Low	13.6

**Table 2:** Family characteristics and environment

Characteristics	%	Characteristics	%
Parent marital status (*n*=965)		Family arrangement (with whom the child currently lives) (*n*=964)	
Living together	91.3	Living with both natural parents	95.1
Divorced	2.7	Living with one natural parent	3.5
Separated	3.5	Not living with natural parent	1.3
Death (one or both)	2.5	Number of siblings (*n*=957)	
Parental education		Alone	8.4
Mother (*n*=946)			
University and college degree	22.2	One	48.6
Technical/vocational education/high school	22.9	More than one	43.1
Completed secondary school	22.7	Parent quarrelling (*n*=965)	
Completed primary school	32.1	Never	42.9
Father (*n*=942)		Rarely	42.6
University and college degree	25.2	Sometimes	12.8
Technical/vocational education/high school	20.4	Often	1.7
Completed secondary school	22.0	Parent fighting (*n*=967)	
Completed primary school	32.5	Never	79.9
Parent occupation		Rarely	15.5
Mother (*n*=954)			
Government staff	18.3	Sometimes	3.8
Self-employed	27.5	Often	0.8
Farmer	33.0	Sibling conflict (*n*=959)	
Housekeeper/unemployment/other	21.2	Never	40.1
Father (*n*=956)		Rarely	29.9
Government staff	24.7	Sometimes	23.1
Self-employed	34.4	Often	6.9
Farmer	30.9	Emotional support (Who do you talk to when you need help?) (*n*=954)	
Housekeeper/unemployment/other	9.7	Father	4.9
Parent alcohol problems (*n*=964)		Mother	20.0
No	89.9	Brother/sister	14.6
Yes	10.1	Friends	36.6
Parent drug problems (*n*=963)		Relatives/others	8.5
No	91.1	No one	15.4
Yes	0.9		

### Prevalence of health risk behaviors by gender, school location, and grade

Between genders, there was one statistically significant difference for ‘consumed alcohol in the past 30 days’, with a larger proportion of males than females consuming alcohol. The differences between the city and suburban schools were also examined. Table 3 shows higher proportions of all risk behaviors in the inner city school compared to the suburban school. Prevalence of driving a motorbike in the past 30 days increased significantly in the higher grades and was highest in grade 8 (about five times more likely than grade 6 and more than two times higher than grade 7) (Table 3).

**Table 3:** Prevalence of health risk behaviors among school children by gender, school location, and grade

Health risk behaviors	Gender			School location			Grade			
		
	Male (*n*=488),%	Female (*n*=465),%	Overall sample (%) (Average)	City school (*n*=496),%	Suburb school (*n*=457),%	Overall sample (Average),%	Grade 6 (*n*=321),%	Grade 7 (*n*=341),%	Grade 8 (*n*=291),%	Overall sample (Average),%
Thought about suicide in the past 12 months	9.9	11.3	10.6	16.1; 95% CI 12.9–19.3	4.6***; 95% CI 2.7–6.5	10.6	9.9	10.6	11.4	10.6
Consumed alcohol in the past 30 days	19.7; 95% CI 16.2–23.2	9.7***; 95% CI 7.0–12.4	14.8	20.3; 95% CI 16.8–23.8	8.3***; 95% CI 5.8–10.8	14.5	13.6	16.3	13.4	14.5
Drove motorbike in the past 30 days	8.5	7.4	8.0	10.1; 95% CI 7.4–12.8	5.7**; 95% CI 3.6–7.8	8.0	2.8; 95% CI 0.9–4.6	6.5; 95% CI 3.9–9.1	15.5***; 95% CI 11.3–19.7	8.0

χ^2^ test comparing prevalence of health risk behaviors among male and female, among students in City School and Suburban School and among students by grade

** *p*<0.01

*** *p<*0.001.

### Factors associated with the health risk behaviors

Significant factors (with odds ratio, or OR, and confidence interval, or CI, presented) associated with each health risk behavior are presented separately by gender in Tables 4, 5, and 6.

**Table 4:** Multivariable logistic analysis of factors associated with thinking about suicide (male and female)

Variables	OR (CI)
Male	
School location	3.9 (1.7–8.7)**
Father care	0.9 (0.8–1.0)**
School connectedness	0.9 (0.8–1.0)*
Bullying	1.2 (1.1–1.4)***
Female	
Economic status	**
High economic status	1.00
Medium economic status	0.4 (0.2–0.8)
School location	3.0 (1.2–7.5)**
Emotional support	**
Mother/father	1.00
Brother/sister (1)	0.9 (0.8–4.8)
Friends (2)	1.7(0.6–4.9)
Relative/others (3)	3.4 (0.8–13.7)
None (4)	2.5 (0.7–9.5)
Mother care	0.9 (0.8–1.0)**
Father over protection	1.1 (1.0–1.2)***
School connectedness	0.9 (0.8–1.0)***

* Note: *p*<0.05

** *p*<0.01

*** *p*<0.001.

**Table 5:** Multivariable logistic analysis of factors associated with drinking alcohol (male and female)

Variables	OR (CI)
Male	
School location	2.2 (1.1–4.6)***
Father education	*
Uni/college degree	1.0
Technical/vocational/high school (1)	0.5 (0.2–1.0)
Complete secondary school (2)	0.3 (0.2–0.7)
Complete primary school (3)	0.6 (0.2–1.5)
Parental alcohol	*
No	1.0
Yes (1)	1.9 (0.9–4.0)
Father care	0.9 (0.8–1.0)**
Siblings conflict	*
Never	1.00
Rarely (1)	2.2 (1.1–4.1)
Sometimes (2)	2.5 (1.2–4.9)
Usually/often (3)	2.8 (1.1–7.2)
Female	
School location	6.3 (2.4–16.6)***
Mother care	0.9 (0.8–1.0)***

* Note: *p*<0.05

** *p*<0.01

*** *p*<0.001.

### Thinking about suicide

For males, Table 4 shows that father care, school connectedness, and bullying were associated with ST. Of those, bullying was associated with an increased risk of ST (OR=1.2), whereas father care and school connectedness were associated with a reduced risk. Inner city school location was also found to be associated with high risk of ST. For females, mother care and school connectedness were associated with a reduced risk of suicidal thoughts, whereas father over-protection had the opposite association. High economic status and seeking emotional support outside of the immediate family (parents or siblings) were also associated with ST among females. Notably, female participants who did not receive emotional support at home had higher risk of ST than those who sought emotional support from mother/father or brother/sister. As in the case of males, inner city school location was also associated with a higher likelihood of ST among female participants (OR=3.0).

### Alcohol consumption by adolescents

Father care and mother care were both associated with a reduced risk of alcohol consumption among males and females. Sibling conflicts were associated with an increased risk of DA among males. Inner city school location was associated with DA in both genders. Males whose fathers had relatively high education (university or college degree) were at the most risk of DA. Males who reported their parents had an alcohol problem were also at risk of DA themselves.

### Underage driving of motorbikes

All adolescents in the survey were under the legal age for driving in Vietnam. For males, mother care was associated with a reduced risk of driving a motorbike. Father occupation was also associated with risk: those males whose fathers were government staff were most likely to report driving motorbikes. Males whose mothers had been educated at technical/vocational/high school, had completed primary school only, or had no education had a higher risk of MD than males whose mothers had a university/college degree. It is clear that the age of male students strongly predicted the risk of underage driving. Among females, mother care was associated with low risk while age was associated with higher risk of driving motorbikes. Female participants who sought emotional support from people other than mother/father or brother/sister were more at risk. Parental alcohol problems also significantly predicted the risk. Inner city school location, again, was associated with the risk of driving motorbikes in females (Table 6).

**Table 6:** Multivariable logistic analysis of factors associated with underage driving motorbike (male and female)

Variables	OR (CI)
Male	
Age	2.8 (1.7–4.6)***
Mother education	*
Uni/college degree	1.0
Technical/vocational/high school (1)	3.0 (1.0–9.0)
Complete secondary school (2)	0.4 (0.9–1.4)
Complete primary school (3)	1.4 (0.4–4.9)
Father occupation	*
Government staff	1.00
Self-employed (1)	0.6 (0.2–1.5)
Farmer (2)	0.2 (0.1–0.8)
Housekeeper/unemployed/others (3)	0.5 (0.1–2.0)
Mother care	0.9 (0.8–1.0)***
Parental fighting	*
Never	1.00
Rarely (1)	2.5 (1.0–6.4)
Often/sometimes (2)	0.000
Female	
Age	1.9 (1.1–3.3)***
School location	7.0 (2.2–21.0)***
Emotional support from	*
Mother/father	1.00
Brother/sister (1)	0.5 (0.1–5.6)
Friends (2)	3.1 (0.9–11.5)
Relative/others (3)	2.6 (0.4–15.5)
None (4)	1.7 (0.3–9.1)
Mother care	0.9 (0.8–1.0)***

* Note: *p*<0.05

*** *p*<0.001.

## Discussion

Recent economic development has enabled Vietnamese children to enjoy better living conditions, but it may also subject them to various risks that negatively influence their health. This study indicates that the prevalence of ST in young people (in the preceding 12 months) is about 11%, and that students in inner city schools are at a significantly higher risk than those in suburban areas (suburban 4.6%; city 16.1%, *p<*0.001). This difference appears to be remarkably large, but it is quite consistent with several other studies in Vietnam, including the national youth surveys (SAVY I and II) (10, 18, 19), and it reflects a pattern in some other Asian countries (5, 20).

Alcohol consumption (past month) was reported by about 15% of the adolescents in this sample, with significant differences by gender and school location, with highest prevalence among inner city males. The estimate for alcohol consumption is higher than that reported in the 9% found in the Vietnamese study conducted by Huong (10) and a prevalence of 8% found by Choo in Malaysia (20). However, all three studies were consistent in terms of the association among drinking, gender, and location.

This study is the first in Vietnam to estimate the prevalence of MD (past month). We have been unable to find other community-based surveys in Asia that examine family and social characteristics of underage drivers. This risk behavior was more prevalent among older, inner city students, although there was no significant difference between males and females. Our data show that maternal care is protective. However, the behavior was not strongly associated with range of family and school variables included in this study. This strongly suggests the need for further research into potentially modifiable factors that influence underage driving in this environment, especially where there is high risk of injury or death from traffic accidents (13).

Children's mental health and risk behavior are influenced by a wide range of factors. This study indicates that for both males and females, school connectedness and parental care were protective factors. Importantly, father care appeared to influence males and mother care influenced females. The findings are generally consistent with the two national surveys (SAVY I and II). The SAVY analysis revealed that positive family relationships and school connectedness correlate with good mental health among Vietnamese youth (21). A study of 1,432 secondary school children aged 12–16 years, examining the relative contribution of parental bonding and peer victimization at school in Adelaide, Australia, by Rigby, Slee and Martin (2007) found that poor mental health in both male and female students was linked with low mother and father care (22). However, that study did not examine the association between parental care and the risk behaviors such as suicidal behaviors and DA. Data from a cross-sectional household survey carried out in six European countries with 7,740 respondents were similar, showing that father and mother care reduced the risk of suicidal ideation (23).

One previously unobserved finding was that mother care had a positive influence on MD in both sexes. From another angle, the SAVY II survey examined the use of motorized vehicles by youth, specifically after drinking, and found that staying in school may be protective against driving under the influence of alcohol (24). Together, these findings suggest that young people with positive mother care and engagement in school may have reduced risk of traffic-related injury and the social consequences of legal violations.

With regard to the negative correlates of behavioral risk, this study found that, while bullying is linked to ST in males, father over-protection had a negative influence on suicidality in females. These findings are similar to research with school children in Adelaide, Australia (22). They found that for both sexes, mother control and father control correlated with anxiety, social dysfunction, and depression, all common precursors of suicidal ideation, In Hong Kong, Lai and McBride-Chang (25) found that parental over-control was associated with high risk of suicidal ideation in both male and female adolescents.

There are some notable points of difference with prior research. The Vietnamese adolescents’ self-reports of parental drug problems, alcohol problems, and divorce had very weak associations with risk behaviors. Also in Vietnam, the SAVY I & II surveys found that there was no association between having a family member with a history of drug or alcohol problem and youth drinking behaviors. This pattern is very different from trends in Western research, where parental drug abuse is often found to have a strong negative influence on their children's behaviors. The difference might be attributed to cultural differences that lead to a lower prevalence of these risk factors, especially very low prevalence of alcohol abuse by mothers, and the relatively low rate of divorce compared to economically developed countries (15).

Inner city school location had a strong influence on behaviors of the school children, especially for girls. There may be a number of contributing factors. Although no associations were found between self-reported (single item) ‘academic achievement’ and any of the three risk behaviors, media reports and other research in Vietnam (26, 27) have highlighted educational stress to be a significant problem in Vietnam. Pressure to succeed academically has negative effects on the well-being and behavior of young people, especially children living in urban areas (18, 19, 21, 26). In inner urban areas, the pressure from parents, teachers, and society may be higher than that in less developed suburban and rural areas. In many families, educational success, especially admission to university, is perceived as essential for their children's future and family pride. Further investigation of the prevalence, indicators, and effects of this problem is needed.

Health risk behaviors need to be recognized and identified early. Therefore, emphasis should be put on increasing capacity for mental health services and on human resource development in this field, including individualized and group support for students, parents, and teachers. For underage driving of motorbikes, there should be involvement not only from the school and family but also enforcement from the government and different sectors such as the police and transportation authorities. The enforcement should be continuous and strong.

### Strengths and limitations of the study

This is one of the few school-based studies on health risk behavior among secondary school children in Vietnam to examine a range of family and school determinants. The interviews were based on standardized measures of behavior and social correlates that have been validated and widely used in similar studies in other countries. The main limitation is that just two schools in Hanoi city participated; they were not randomly selected and therefore the results may not be representative of other cities and rural areas. As with many other studies of this type, all data were gathered by retrospective self-report, so there may have been recall bias that reduced the accuracy of the findings.

## Conclusion

This study revealed a number of factors that influence risk behaviors among Vietnamese school students. Findings of importance to potential intervention programs are that school connectedness and high quality parental care have positive influences, whereas bullying, family conflict, and parental over-protection appear to increase risks to health. The problem behaviors were especially common among young people in inner urban areas.

To prevent young people from MD, it is suggested that general population awareness campaigns should emphasize good parental (particularly maternal) communication about traffic safety, focusing on adolescents aged 15 or 16 years. Involvement from the school and family is important, but enforcement by the government and different sectors, such as police and transportation authorities, is needed to prevent school children from driving motorbikes. With regard to prevention of suicide, it is recommended to improve school connectedness among children and implement programs that have been proven in other countries to reduce bullying. These initiatives could be implemented effectively through health promotion approaches in schools. More in-depth study in this field is necessary to expand the evidence base for action that is urgently needed to promote mental health and well-being among young people in Vietnam.
